# Correction: Droplet microfluidics: fundamentals and its advanced applications

**DOI:** 10.1039/d0ra90086a

**Published:** 2020-09-03

**Authors:** Somayeh Sohrabi, Nour Kassir, Mostafa Keshavarz Moraveji

**Affiliations:** Department of Chemical Engineering, Amirkabir University of Technology, Tehran Polytechnic Iran moraveji@aut.ac.ir

## Abstract

Correction for ‘Droplet microfluidics: fundamentals and its advanced applications’ by Somayeh Sohrabi *et al.*, *RSC Adv.*, 2020, **10**, 27560–27574, DOI: 10.1039/D0RA04566G.

The authors regret the omission of a funding acknowledgement in the original article. This acknowledgement is given below.

The authors would like to acknowledge the financial support of the Iran National Science Foundation (INSF), grant number 98017171.

In addition, the authors regret that incorrect reference numbers were given in Table 1 of the original article. The corrected table and references are shown below.

**Table 1:** Size and frequency distributions for various droplet generation systems

	Geometry and material	Continuous phase	Size/μm	Frequency/Hz	Ref. in original article reference list	Ref. in this Correction
Water in oil	Channel array in silicon	Kerosene with monolaurate	21	∼5300 (est.)	—	1
	T-junction in acrylated urethane	Decane, tetradecane, and hexadecane with Span 80	10 to 35	20 to 80	—	2
	T-junction in PMMA	High oleic sunflower oil	100 to 350	10 to 2500	—	3
	T-junction in PDMS	C_14_F_12_ with (C_6_F_13_)(CH_2_)_2_OH	7.5 nl (plug flow)	2	55	4
	Shear-focusing in PDMS	Oleic acid	13 to 35 (satellites <100 nm)	15–100	49	5
Oil in water	Channel array in silicon	Water with SDS	22.5	∼5300 (est.)	—	1
	Sheath flow in glass capillary	Water with SDS	2 to 200	100 to 10 000	—	6
Gas in liquid	Flow-focusing in PDMS	Water with Tween 20	10 to 1000	>100 000	—	7
	Shear-focusing in PDMS	Water with phospholipids	5 to 50	>1 000 000	—	8
Liquid in air	DEP on hydrophobic insulator	Air	10 pl	∼8 (est.)	57	9
	EWOD on hydrophobic insulator	Air	∼700 nl	∼1 (est.)	28	10

The Royal Society of Chemistry apologises for these errors and any consequent inconvenience to authors and readers.

## Supplementary Material
